# Gut microbiota: Linking nutrition and perinatal depression

**DOI:** 10.3389/fcimb.2022.932309

**Published:** 2022-08-26

**Authors:** Jia Song, Bi Zhou, Juntao Kan, Guangya Liu, Sheng Zhang, Liang Si, Xianping Zhang, Xue Yang, Junhua Ma, Junrui Cheng, Xiaobo Liu, Yongde Yang

**Affiliations:** ^1^ Affiliated Wuhan Mental Health Center, Tongji Medical College, Huazhong University of Science and Technology, Wuhan, China; ^2^ Nutrilite Health Institute, Shanghai, China; ^3^ Jinyintan Hospital, Wuhan, China; ^4^ Ingredion Incorporated, Bridgewater, NJ, United States

**Keywords:** fiber, perinatal depression, nutrition, microbiota, probiotics

## Abstract

Perinatal depression is a mood disorder that is reported in women during pregnancy (prenatal) and after childbirth (postnatal). The onset of perinatal depression is associated with changes in reproductive hormones, stress hormones and neurosteroids. These chemical compounds can be modulated by the gut microbiota, which may affect maternal mental health during the perinatal period *via* the gut-brain-axis. Recent studies suggest that nutritional and dietary interventions (vitamin D, ω-3 fatty acids, iron, and fiber) effectively prevent or mitigate maternal depression and anxiety, but their efficacy is confounded by various factors, including the gut microbiota. Probiotics are efficacious in maintaining microbiota homeostasis, and thus, have the potential to modulate the development of perinatal mood disorders, despite no evidence in human. Therefore, clinical trials are warranted to investigate the role of probiotic supplementation in perinatal depression and behavioral changes. This article reviews the interplay between nutrition, gut microbiota and mood and cognition, and the evidence suggesting that probiotics affect the onset and development of perinatal depression.

## Introduction

Perinatal depression is defined as the onset of a major or minor depressive episode during pregnancy (antenatal depression), after childbirth (postpartum depression), or both (Leung and Kaplan, 2009). Owing to the changes in circulating biochemicals, mood disturbances have been reported in up to 80% mothers within few days of delivery (Van Niel and Payne, 2020). If untreated, perinatal depression may result into serious complications, such as premature birth, preeclampsia, maternal morbidity, and even maternal suicide (Jarde et al., 2016). Therefore, it is critical for the general public to raise awareness about perinatal depression.

The human gut harbors trillions of microorganisms that form a complex and dynamic ecosystem (Clemente et al., 2012). The gut microbiome is a crucial factor that shapes and modulates immune responses, indicated by the link between gut microbial dysbiosis and multiple diseases (Li et al., 2018; Kan et al., 2020; Metwaly et al., 2022). In addition, the gut microbiota affects mood, cognition and brain health by regulating nervous, endocrine, and immune signaling mechanisms (Dinan and Cryan, 2017) because of its ability to modulate the activity of the hypothalamic-pituitary-adrenal (HPA) axis. Furthermore, an altered gut microbial profile has been strongly associated with anxiety and depression (Bear et al., 2020). However, dietary and nutritional interventions have potent effects on reducing depression (Yang et al., 2020), where microbiota may serve as important mediators (Dahl et al., 2020).

Probiotics include live bacteria and/or yeast intended to maintain or improve the normal microflora in the gut. Although probiotic supplements are increasingly consumed during pregnancy to enhance maternal health, their effects on perinatal depression have not been reviewed. Therefore, the objective of this article was to review the role of microbiota on the interactions between diet, nutrition, and maternal mental health, and the mechanism by which probiotic intake may alleviate perinatal depression.

## Nutrition and perinatal depression

### Vitamin D

The prevalence of vitamin D deficiency was prevalent in women during pregnancy and in newborns is a serious concern (Bodnar et al., 2007; Dijkstra et al., 2007; Lee et al., 2007), because vitamin D is associated with hippocampal learning and memory in mothers and neural cell growth in the offspring (Liang et al., 2018; Li et al., 2021). Previous studies suggest that vitamin D acts as a neuroactive hormone and modulates the concentration of neuronal calcium ions, which regulate neuroplasticity and mood (de Abreu et al., 2009; Berridge, 2017). A meta-analysis of nine longitudinal studies with 8,470 subjects revealed a significantly inverse association between serum 25(OH)D levels and the risk of postpartum depression, with a cutoff of 50 nmol/L (Wang et al., 2018). Nonetheless, a randomized controlled trial indicated that supplementation with 2000 IU vitamin D_3_ from 26-28 weeks of gestation up to childbirth significantly reduced depression scores, suggesting that vitamin D supplementation during late pregnancy was beneficial for diminishing perinatal depression.

### ω-3 fatty acids

Several *in vivo* studies suggest that ω-3 fatty acids (FA) may modulate depression. Pusceddu et al. showed that supplementation of eicosapentaenoic acid (EPA) and docosahexaenoic acid (DHA) effectively restored altered gut microbiota induced by maternal separation-related stress (Pusceddu et al., 2015). In contrast, ω-3 FA deficiency in pregnant mice resulted in increased depression and fear-induced freezing behavior in their offspring, indicating that maternal ω-3 FA availability may affect neurobehavioral and cognitive development in the fetus (Robertson et al., 2017). Two meta-analyses have reported that ω-3 FA supplementation reduced the symptoms of depression in patients with mood disorders, but exhibited limited effects on normal populations (Appleton et al., 2010; Grosso et al., 2014). Mechanistic studies showed that both EPA and DHA can prevent inflammation-induced reduction in neurogenesis and apoptosis, which is mediated by lipoxygenase and cytochrome P460 EPA/DHA metabolites in hippocampal neurons (Borsini et al., 2021).

### Iron

Evidence suggest that iron deficiency is associated with psychiatric morbidity, including depression. A storehouse of iron, the basal ganglia in the brain responds rapidly to abnormal iron levels, which, in turn, affects the ability of the brain to process emotions. A recent study revealed that patients suffering from iron-deficiency anemia exhibited significantly higher incidence of sleep disorders, anxiety disorders, and depression compared to healthy controls (Lee et al., 2020). Similarly, Shariatpanaahi et al. reported that the odds of depressive symptoms was significantly increased in patients with low ferritin levels compared to healthy ones (Vahdat Shariatpanaahi et al., 2007).

### Fiber

Dietary fiber is a vital component of healthy diet. A meta-analysis of four case-control studies indicated that compared to healthy controls, patients with depression exhibited significantly lower fiber intake. This investigation also pooled five cross-sectional studies and revealed that dietary fiber consumption was inversely associated with the odds of depression (Fatahi et al., 2021). However, these observational studies did not determine the causality of fiber intake and the onset of depression. Moreover, the benefits of fiber consumption against perinatal depression were increasingly evident in obese rats, whose fiber-enriched diets significantly attenuated cognitive effects and maternal depression (Liu et al., 2020). In addition, an increased dietary fiber intake has been associated with reduced depression in premenopausal, but not in postmenopausal women (Kim et al., 2021).

Fructo-oligosaccharides (FOS) and galacto-oligosaccarides (GOS) are considered as prebiotics as they selectively elevate the populations of health-promoting bacteria, whereas other fibers, which facilitate the growth of all gut bacteria, including pathogenic bacteria, are not deemed as prebiotics (Gibson et al., 2017). Inulin-type FOS have been implicated in the inhibition of mild stress-induced depression and intestinal epithelial damages by lowering corticosterone levels (Chi et al., 2020). In humans, supplementation of GOS effectively mitigated anxiety, but the effects were not clinically significant (Johnstone et al., 2021). Therefore, clinical trials are required to validate the efficacy of FOS and GOS in alleviating stress and mood disorders.

## Nutrition and microbiota

The gut microbial profile can be modulated by altering lifestyle, such as nutrition and diet. As mentioned previously, the intake of vitamin D, ω-3 FA, and fiber intakes has been associated with depression. Notably, these nutrients and fiber are also involved in modulating the gut microbiota, which may consequently mediate their efficacy as anti-depressants (**Table 1**).

**Table 1:** The effects of nutrients on the gut microbiota and depression.

Nutrients	Microbiota Genus	Change with supplementation	Increase or decrease in depressive patients	Overall effect on depression
Vitamin D	*Akkermansia*	Inhibit	Increase	Ameliorate
	*Bifidobacteria*	Enhance	Decrease	Ameliorate
	*Coprococcus*	Enhance	Decrease	Ameliorate
	*Erysipelotrichaceae*	Decrease	Increase	Ameliorate
	*Lactococcus*	Enhance	Unknown	Unknown
	*Porphyromonas*	Decrease	Increase	Ameliorate
	*Ruminococcus*	Decrease	Unknown	Unknown
	*Veillonella*	Decrease	Increase	Ameliorate
ω-3 PUFA	*Bifidobacterium*	Enhance	Decrease	Ameliorate
	*Lactobacillus*	Enhance	Decrease	Ameliorate
	*Roseburia*	Enhance	Decrease	Ameliorate
Fiber (FOS, GOS)	*Anaerostipes*	Inhibit	Increase	Ameliorate
	*Bifidobacterium*	Enhance	Decrease	Ameliorate
	*Cyanobacteria*	Enhance	Unknown	Unknown
	*Lactobacillus*	Enhance	Decrease	Ameliorate
	*Oscillibacter*	Inhibit	Unknown	Unknown
	*Proteobacteria*	Inhibit	Unknown	Unknown
	*Ruminococcus*	Inhibit	Unknown	Unknown
Iron	*Bifidobacteria*	Inhibit	Decrease	Promote
	*Lactobacillus*	Inhibit	Decrease	Promote

Vitamin D and ω-3 FA deficiencies have been linked to gut dysbiosis and gastrointestinal (GI) inflammation, where both vitamin D and ω-3 FA can enhance gut microbial diversity and decrease the Firmicutes to Bacteroidetes ratio (F/B ratio) (Robertson et al., 2017; Singh et al., 2020). In addition, vitamin D and ω-3 FA supplementation may promote the abundance of *Bifidobacterium*; vitamin D intake may increase the abundance of *Akkermansia* (Charoenngam et al., 2020; Singh et al., 2020), *Coprococcus* (Naderpoor et al., 2019), and *Lactococcus* (Kanhere et al., 2017) and decrease the abundance of *Porphyromonas* (Charoenngam et al., 2020), *Ruminococcus* (Naderpoor et al., 2019), *Veillonella* and *Erysipelotrichaceae* (Kanhere et al., 2017), whereas ω-3 FA may increase the abundance of *Lactobacillus* and decrease the abundance of *Anaeroplasma*, *Clostridium*, and Peptostreptococcaceae members, as shown in an *in vivo* study (Robertson et al., 2017), and enhance *Bifidobacterium*, *Lactobacillus*, and *Roseburia*, as shown in a clinical trial (Watson et al., 2018). The role of Firmicutes has been well-documented in the development of depression. An *in vivo* study reported that reduced F/B ratio was associated with depression-like behaviors in mice that underwent chronic stress (Bailey et al., 2011). Similarly, decreased abundance of *Firmicutes* has been associated with the progression of depression (Naseribafrouei et al., 2014; Kelly et al., 2016; Jiang et al., 2018). Moreover, lipopolysaccharide (LPS) derived from the gut microbiota are considered as major endotoxins that can trigger inflammation and systemic endotoxemia (Lin et al., 2020). Nevertheless, LPS play a complicated role in depression. Although LPS overproduction can lead to inflammation, two studies have reported that LPS produced by gut bacteria might promote immunity in depressed patients (Maes et al., 2010; Maes et al., 2012). Therefore, further studies are necessary to determine the role of LPS homeostasis in depression.

Compared to iron surplus, iron deficiency exhibited increased influence on gut microbiota dysbiosis (Rusu et al., 2020). According to a clinical trial on children, iron supplementation at a high dosage of 50 mg per day (4 days/week) for 38 weeks did not alter short chain fatty acids in the feces (Dostal et al., 2014). However, iron supplementation may increase the abundance of Enterobacteriaceae members such as the pathogenic strains of *Escherichia coli*. In addition, iron surplus may reduce the abundance of *Bifidobacterium* and *Lactobacillus* (Paganini and Zimmermann, 2017), whereas iron deficiency may decrease the Bifidobacteriaceae/Enterobacteriaceae ratio (Muleviciene et al., 2018). Therefore, a balanced iron intake is critical to maintain normal abundance of Bifidobacteria to address depression (Altaib et al., 2021).

Dietary fibers are either polysaccharides with a minimum of 10 sugar moieties or oligosaccharides with 3-10 sugar residues. Although fiber cannot be metabolized by humans, they can be utilized by certain species in the gut microbiota through anaerobic fermentation, thereby modulating the gut microbial profile. The final products of anaerobic fermentation include short chain fatty acids (SCFA), namely acetate, propionate, and butyrate. The supplementation of FOS resulted in reduced levels of depression-associated bacteria (e.g., *Anaerostipes*, *Oscillibacter*, *Proteobacteria*, and *Streptococcus*) and increased the abundance of the phylum Cyanobacteria, which is a group of bacteria that can produce H_2_S, a metabolite that exhibits antidepressant-like properties (Chi et al., 2020). Moreover, both FOS and GOS can promote the abundance of *Bifidobacterium* (Liu et al., 2017), while the effect of FOS in affecting butyrate-producing microbes, including *Phascolarctobacterium* and *Ruminococcus* were inconsistently reported (Liu et al., 2017; Tandon et al., 2019).

In addition to single nutrients or nutritional compounds, dietary patterns exhibited significant association with depressive symptoms. Poor diets have been reported to significantly alter the abundance and diversity of gut microbes. Several studies also suggest pathophysiological links between unhealthy diet and susceptibility of depression. Additionally, dietary patterns characterized by high consumption of processed meat, refined carbohydrates, simple sugars, high-fat dairy products (e.g., butter), and gravy and low consumption of fruits and vegetables are positively correlated with the risk of depression (Li et al., 2017). Such dietary patterns exhibit major features of a western diet that contains excessive saturated fat, simple sugars, salt, and food additives. In contrast, a multicenter, randomized, primary prevention field trial on patients with type 2 diabetes revealed that a Mediterranean diet supplemented with nuts a significantly reduced the risk of depression (Sánchez-Villegas et al., 2013).

## Mechanisms of perinatal depression

### Reproductive hormones

Using positron emission tomography and functional magnetic resonance imaging, researchers have revealed that gonadal steroids hormones modulate neurocircuitry, neuroplasticity, and cortical activity during normal and pathological affective states (Rubinow and Girdler, 2011; Schiller et al., 2015). Estrogen is an ovarian hormone that can modulate serotonergic functions by regulating tryptophan hydroxylase and serotonin reuptake transporter in raphe nuclei (Shors and Leuner, 2003). Additionally, reduced serotonin levels are associated with depression and arousal. The administration of an estrogen receptor (ER) antagonist to hippocampus resulted in anxiety and depression behavior in female rats. Once activated, ERs, such as ERα and Erβ, may display anxiolytic-like effects, but the onset of postpartum depression has been associated only with changes in ERα and ERα-related system (Furuta et al., 2013). In women with low estrogen levels, estrogen administration may improve mood and alleviate depressive symptoms (Gregoire et al., 1996; Ahokas et al., 2001). Nevertheless, several risks and adverse effects, such as increased production of coagulation factors and pro-inflammatory cytokines, hypertriglyceridemia, venous thromboembolism, and gallstones, are associated with long-term estrogen administration (Mehta et al., 2021).

Similar to estrogen, progesterone is a critical hormone for sexual and reproductive development in females. During pregnancy, progesterone facilitates uterus growth and lactation. Studies have also shown the ability of progesterone to stimulate GABA and brain-derived neurotrophic factor (BDNF) in the hippocampus and cerebral cortex. However, in a study by Osborne et al., progesterone levels in the second or third trimesters were not associated with the risk of postpartum depression (Osborne et al., 2017). Considering the small sample size of this study, these observations could not be verified. Thus, further large-scale studies on the role of progesterone in postpartum depression are warranted.

Oxytocin, a hormone produced in the hypothalamus and involved in childbirth and lactation, exhibited a significant inverse relationship with postpartum depression (Thul et al., 2020). Mechanistically, oxytocin modulates depression by activating a MAP kinase (MAPK) cascade, subsequently phosphorylating cAMP response element-binding protein (CREB), leading to enhanced BDNF expression (Matsuzaki et al., 2012). Furthermore, enhanced estrogen and progesterone levels increase oxytocin transcription in hypothalamus, which primarily regulates maternal behavior and lactation (Schiller et al., 2015).

### Stress hormones

Cortisol, a glucocorticoid, is a key physiological inducer of stress response (Lancaster et al., 2010). In a study on 44 pregnant women, researchers observed that the hair cortisol levels during the first and third trimesters were positively correlated with pregnancy-related stress, indicating that hair cortisol levels could serve as a potential predictor or indicator of maternal depression (Caparros-Gonzalez et al., 2017). However, cortisol secretion depends on corticotropin releasing factor (CRF), which regulates the response of the HPA axis and is produced in response to real or imagined cognitive stressors (Seth et al., 2016). Moreover, CRF can stimulate the release of adrenocorticotropic hormone (ACTH), leading to the production of cortisol in the adrenal cortex. Since human placenta contains biologically active CRF, hypothalamic CRF production is increased during pregnancy, resulting in high levels of cortisol and hypercortisolemia.

### Neurosteroids

Neurosteroids refer to endo- and exogenous steroids produced in the brain to regulate neuronal excitability by regulating nuclear steroid receptors (Reddy, 2010). They also play an important role in the development of mood disorders, cognitive disfunctions, and memory deficits (Ratner et al., 2019). Allopregnanolone (3α-hydroxy-5α-pregnan-20-one) is a progesterone metabolite that can enhance GABAergic signaling by interacting with the GABA_A_ receptor. Low levels of allopregnanolone have been associated with increased risk of mood disorders, including depression during late pregnancy (Hellgren et al., 2014). Typically, the concentration of serum allopregnanolone ranges from 0.5-5 nmol/L, varying with the stages of the menstrual cycle. However, serum allopregnanolone concentration first increases to more than 10 times during the antenatal period, and then rapidly decreases to 1-2 nmol/l after parturition (Nappi et al., 2001; Pearson Murphy et al., 2001), suggesting a potential mechanism of postnatal depression.

### Inflammation

Women at different trimesters of pregnancy exhibit distinct inflammatory profiles, with the first, second, and third being majorly pro-inflammatory, anti-inflammatory, and pro-inflammatory states, respectively (Mor and Cardenas, 2010). Individuals responds to inflammatory cytokines vary based on history of depression, medical history, body mass index, and psychosocial status (Sawyer, 2021). According to large-scale meta-analyses, higher levels of IL-6, IL-12, IL-13, IL-18 and TNFα were observed in patients with depression (Köhler et al., 2017a). A prospective study with a follow-up period of 12 years also suggested a predictive role of proinflammatory cytokines including C-reactive protein (CRP) and IL-6, in depression (Gimeno et al., 2009). These findings were conspicuous because pro-inflammatory cytokines have been reported to affect serotonin metabolism, increase neurodegeneration by impairing BDNF release, and interfere with monoamine, glutamate and neuropeptide metabolism, resulting in altered neurotransmitter levels and the development of mood disorders (Felger and Lotrich, 2013).

## Microbiota affects depression level *via* the microbiota-gut-brain-axis

The microbiota-gut-brain-axis (MGBA) was identified based on the findings that linked the gastrointestinal endocrine system with alterations in neurons and brain cells (Track, 1980). Subsequently, this concept was supported by studies that reported the contribution of gut microbiota to the development of various cognitive diseases (Chang et al., 2022). Significant differences have been observed in the gut microbial profile of patients with depression and healthy controls (Naseribafrouei et al., 2014; Jiang et al., 2018). Moreover, disturbances in cognition and mood have been reported in patients with gut inflammation (Masand et al., 1995; Addolorato et al., 1997; Kurina et al., 2001). Previous *in vivo* studies revealed that GI inflammation induced anxiety-like behavior (less exploration and more behavioral inhibition) and altered the central nervous system (Lyte et al., 1998; Bercik et al., 2010). To determine causality, researchers leveraged germ-free (GF) animals and observed that the absence of gut microbiota promoted anxiety and neuroendocrine response to a stressful environment (Desbonnet et al., 2008; Crumeyrolle-Arias et al., 2014). Compared to GF mice, gnotobiotic mice with normal specific pathogen-free microbiota were less anxious and were more active (Neufeld et al., 2011; Nishino et al., 2013). Well-designed prospective studies have also depicted the impact of dysbiosis on mental health disorders. In both adults and infants, dose- and time-dependent associations have been reported between antibiotic usage and depression (Lurie et al., 2015; Köhler et al., 2017b; Slykerman et al., 2017b), indicating that alterations in gut microbiota may have a substantial impact on mental health disorders or behavioral changes throughout life.

Intriguingly, studies have shown that depressive symptoms can be transmitted to recipients of fecal microbiota transplantation (FMT). In a study by Zheng et al., after transplanting the fecal microbiota of depressed individuals and healthy controls in GF mice, the recipients of fecal microbiota of depressed patients exhibited more severe depressive symptoms compared to those who received the fecal microbiota of healthy subjects (Zheng et al., 2016). Similarly, Kelly et al. conducted FMT by oral gavage of the fecal microbiota from depressed patients or controls in a microbiota-deficient rat model, and reported that the recipients of fecal microbiota from depressed patients developed more severe anhedonia and anxiety-like behavior, with disrupted tryptophan metabolism. However, these alterations were not observed in the recipients of fecal microbiota from healthy controls (Kelly et al., 2016). These findings demonstrated a causal role of gut microbiota in the development of depressive symptoms.

**Figure 1:**
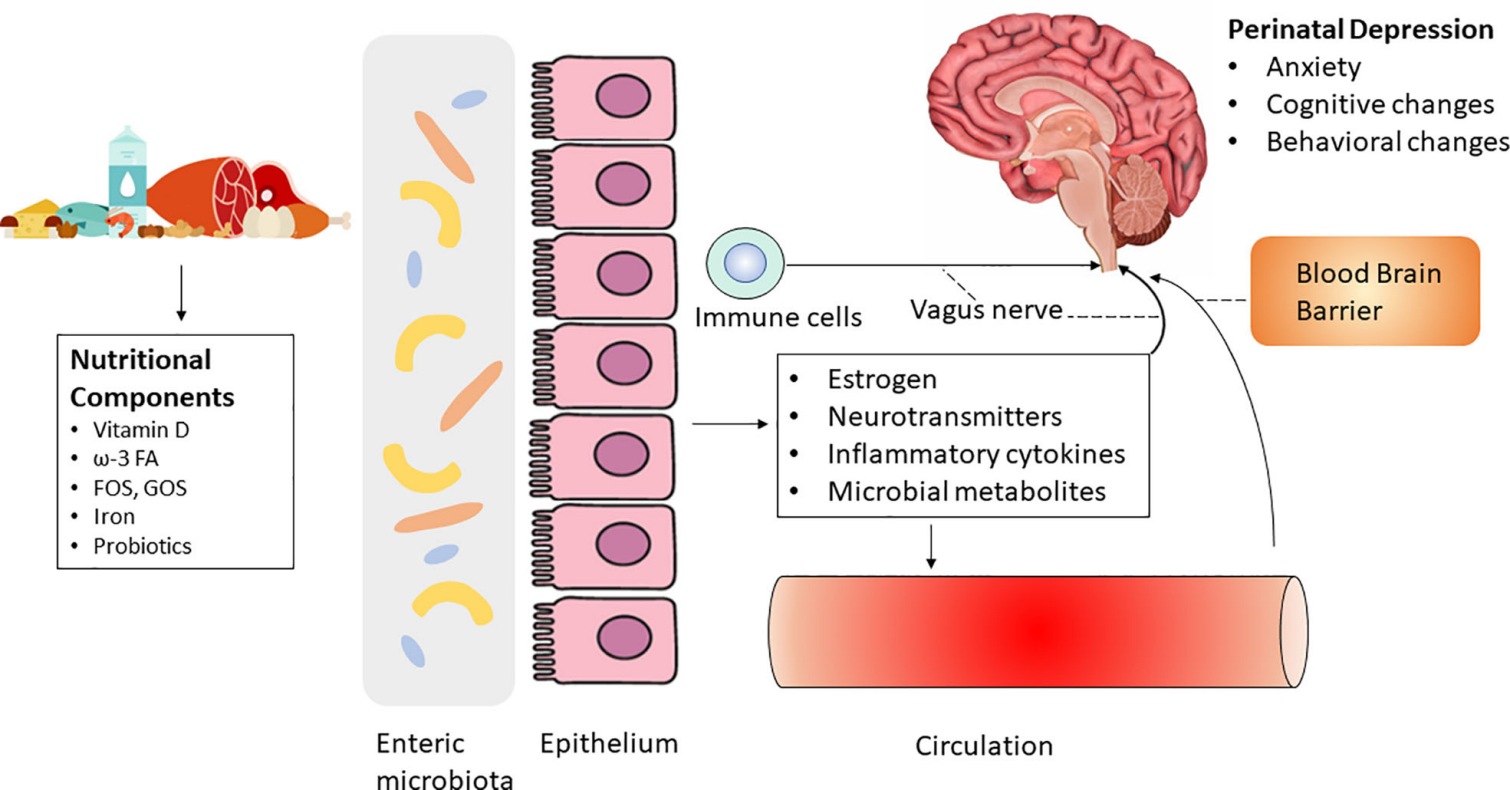
Diet and nutrition may regulate perinatal depression *via* the microbiota-gut-brain axis by influencing systemic biomarkers including estrogen, neurotransmitters, inflammatory cytokines and microbial metabolites. The role of probiotics on perinatal depression is controversial.

Mechanistically, the abundance and profile of microbiota greatly influence GI health by modulating the epithelial layer and mucosa (Valdes et al., 2018). Several factors, such as medication, infection, and lifestyle (e.g., diet), can substantially alter the composition of the gut microbiota, thereby affecting GI health and the progression of various diseases (Cheng et al., 2021; Durmusoglu et al., 2021). As the gut microbiota and brain communicate through multiple routes, including the MGBA. MGBA is gaining increased attention in fields investigating mood and cognition disorders because the gut microbiota is linked to hormonal changes, which may be implicated in psychiatric disorders (Cryan et al., 2019) **(** **Figure 1** **)**.

### Estrogen

**Figure 2:**
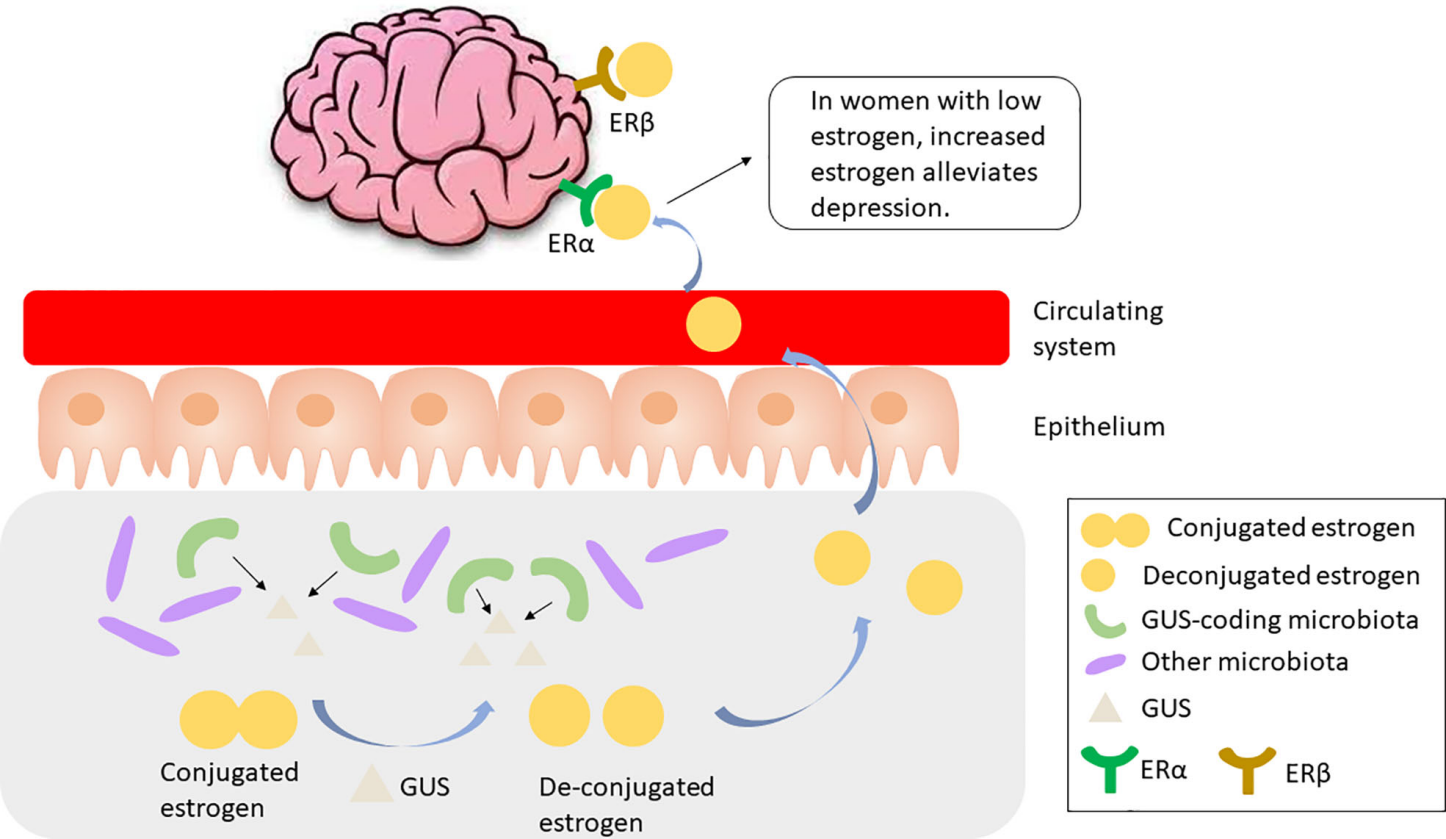
GUS-coding microbiota produces GUS to cleave and activate conjugated estrogen, which is released to the circulating system and binds to ERα, resulting in alleviated depression in women with low estrogen. GUS, β-glucuronidase; ER, estrogen receptor.

The gut microbiota has been implicated in the regulation of circulating estrogens. Mechanistically, gut microbiota regulates estrogen by secreting β-glucuronidase (GUS), an enzyme that deconjugates estrogens into their active forms (Baker et al., 2017). Till date, more than three thousand total and 279 unique microbiome-encoded microbiome-encoded β-glucuronidase (GUS) enzymes have been identified (Sui et al., 2021). However, only 60 bacterial genera in the human GI tract encode GUS, including *Bifidobacterium*, *Lactobacillus*, *Roseburia*, and *Clostridium* (Kwa et al., 2016). The microbial species that encode GUS or GUS candidates have been detailed in (Sui et al., 2021). The component of the gut microbiota expressing GUS is particularly important as these species play a role in recycling inactivated GUS by either metabolizing it and releasing the metabolites into the circulating system, or releasing the parent compound into the GI lumen **(** **Figure 2** **)**. Thus, disruption in the gut microbiome, or dysbiosis, may reduce circulating estrogens, resulting in estrogen-mediated pathologies including cognition deficits (Sherwin, 2012).

### Neurotransmitters

Various neurotransmitters such as GABA, serotonin, glutamate, histamine, and acetylcholine (Ach), are synthesized by the gut microbiota (Oleskin et al., 2016). Several Lactobacillus species produce a wide range of neurotransmitters (Yong et al., 2019) **(** **Table 2** **)**. GABA can be synthesized by certain Bifidobacterium species (Barrett et al., 2012), *Bacteroides fragilis* (Strandwitz et al., 2019), and *L. plantarum* (An et al., 2021). Furthermore, microbial species, including *L. plantarum*, *B. subtilis*, and *S. aureus*, can produce Ach (Horiuchi et al., 2003; Koussoulas et al., 2018; O’Donnell et al., 2020). In addition, Staphylococcus can synthesize and release tyramine, serotonin and tryptamine (Luqman et al., 2018) **(** **Table 2** **)**. As neurotransmitters are transported between neurons for communicating and regulating cognition and emotion, microbiota can promote the crosstalk between the gut and brain by producing these chemical messengers.

**Table 2:** Neurotransmitter producing bacteria.

Microbiota		Neurotransmitter released	Reference
Genus	Species		
Bacillus	*B. Subtilis*	Acetylcholine	(Tsavkelova et al., 2000)
		Dopamine	
		Noradrenaline	
Bacillus	*B. mycoides*	Noradrenaline	(Tsavkelova et al., 2000)
Bacteroides	*B. fragilis*	GABA	(Strandwitz et al., 2019)
Bifidobacterium	*B. dentium*	GABA	(Barrett et al., 2012)
Bifidobacterium	*B. longum subsp. infantis*	GABA	(Barrett et al., 2012)
Bifidobacterium	*B. adolescentis*	GABA	(Barrett et al., 2012)
Lactobacillus	*L. brevis*	GABA	(Barrett et al., 2012)
Lactobacillus	*L. bulgaricus*	Dopamine	(Mazzoli and Pessione, 2016)
		Glutamate	
		Norepinephrine	
Lactobacillus	*L. casei*	GABA	(Yokoyama et al., 2002; Yong et al., 2019; Sharafi and Nateghi, 2020)
		Dopamine	
		Glutamate	
		Norepinephrine	
Lactobacillus	*L. helveticus*	Dopamine	(Oleskin et al., 2014)
		GABA	
		Glutamate	
		Norepinephrine	
		Serotonin	
Lactobacillus	*L. paracasei*	GABA	(Komatsuzaki et al., 2008)
		Glutamate	
Lactobacillus	*L. plantarum*	Acetylcholine	(Stanaszek et al., 1977; Özogul, 2011; Özoğul et al., 2012; Yong et al., 2019)
		Dopamine	
		GABA	
		Glutamate	
		Histamine	
		Serotonin	
Lactobacillus	*L. reuteri*	GABA	(Pokusaeva et al., 2017)
Lactobacillus	*L. rhamnosus*	GABA	(Siragusa et al., 2007)
Serratia	*S. marcescens*	Dopamine	(Tsavkelova et al., 2000)
		Noradrenaline	
Staphylococcus	*S. aureus*	Dopamine	(Tsavkelova et al., 2000)
Noradrenaline			

### Inflammation

The gut microbiota plays an important role in inflammatory diseases by modulating the levels of pro- and anti-inflammatory cytokines. Although microorganisms are primarily confined to the gut by the epithelial barrier, microbial metabolites can penetrate the barrier and enter this circulating system. Notably, increased circulating LPS may result in enhanced levels of TNFα, MCP-1, IL-1β, and NF-κB p65 in the brain, resulting in neuroinflammation, neurodegeneration, and behavioral changes (Qin et al., 2007). An elevated level of circulating proinflammatory cytokines may compromise the blood-brain barrier (BBB), and long-term exposure to these pro-inflammatory cytokines may also lead to neuropsychiatric disorders and depression (Felger and Lotrich, 2013). Evidence from rodent experiments explain the mechanism by which microbiota affects depression by altering inflammatory markers. Zhang et al. reported that stressed mice exhibited significantly more severe oscillopsia and reduced F/B ratio. Nonetheless, intracerebroventricular administration of an anti-mouse IL-6 receptor antibody (MR16-1) effectively restored F/B ratio and improved oscillopsia, suggesting that the interaction between inflammatory cytokines and microbiota was associated with depressive symptoms (Zhang et al., 2017). Compared to the control group, depressive patients treated with antidepressant exhibited substantially decreased Caspase-1 levels (Alcocer-Gómez et al., 2014), which cleaves IL-1β. The loss of Caspase-1 activity increased the abundance of *Akkermansia* spp. and restabilized the gut microbiota profile. Therefore, modulating the gut microbiota by blocking inflammatory cytokine receptors may serve as a promising therapeutic approach for depression.

**Figure 3:**
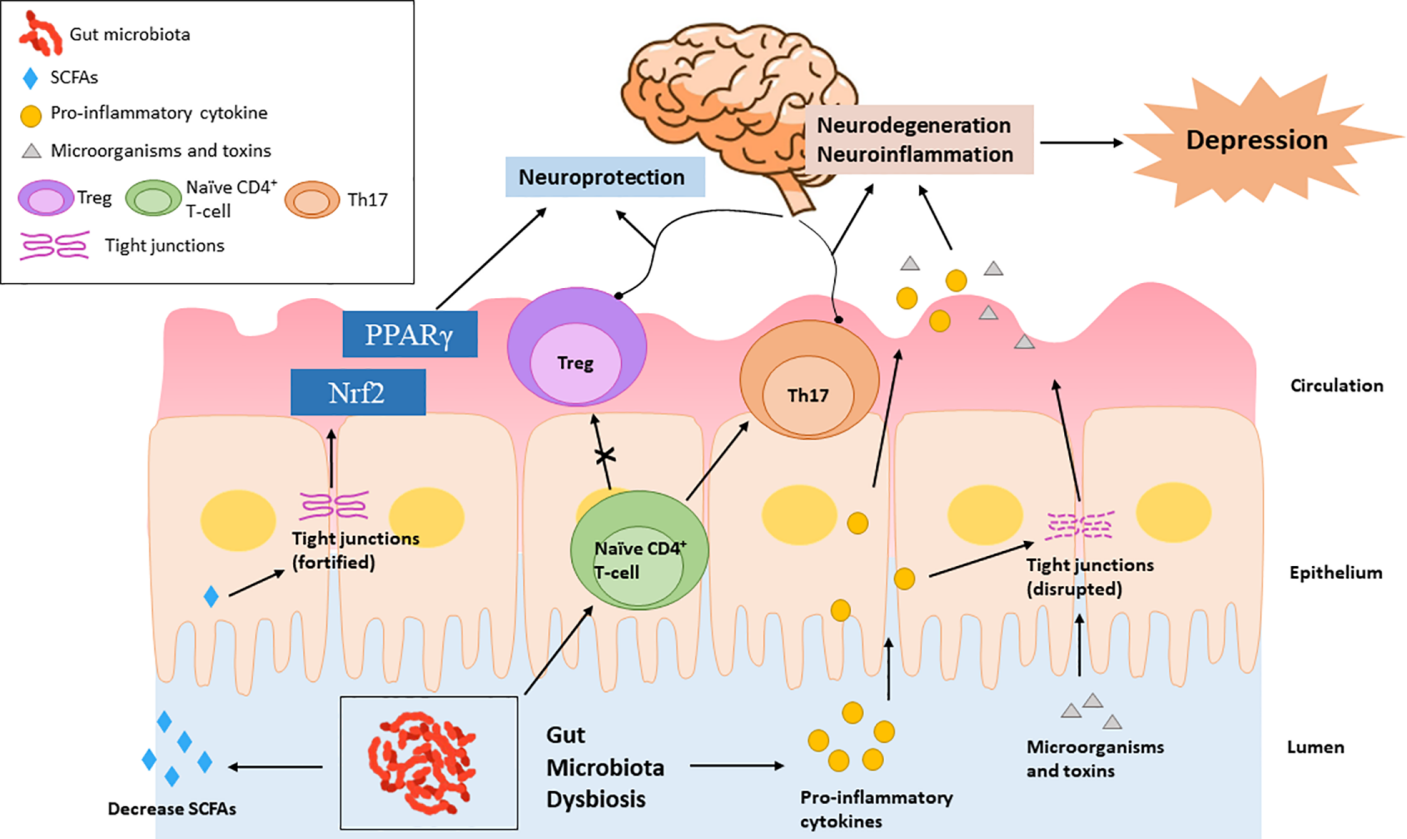
Microbiota dysbiosis increases pro-inflammatory cytokines, which promote the differentiation of naïve autoreactive CD4^+^ T-cells in Th17 cells and subsequently cause neurodegeneration. Elevated pro-inflammatory cytokines can also impair the tight junctions, leading to the intestinal transport of microorganisms and bacterial toxins, causing neuroinflammation. Microbiota dysbiosis decreases SCFAs, which exhibit neuroprotective functions by enhancing the intestinal tight junction and up-regulating the PPARγ and Nrf2 signaling pathways.

Peripheral insults that result in systemic inflammation may facilitate the recruitment of peripheral immune cells to the brain and cause the overactivation of neuroimmune response, leading to neurodegenerative diseases and mood disorders (Cheng et al., 2018; Wendeln et al., 2018; Peng et al., 2022). Schiweck et al. reported a higher level of circulating T helper (Th) cells, especially Th17 cells, in the patients with severe depression (Schiweck et al., 2020). Th17 cells mainly present and polarize in the gut, exhibiting high plasticity. Microbiota dysbiosis has been reported to promote the differentiation of CD4^+^ T-cells into Th17 cells and their pathogenicity (Shao et al., 2020; Su et al., 2020). Although an imbalanced Th1/Th2 ratio, with increased Th1 phenotype, was observe in depressive patients (Myint et al., 2005), the induction of Th1 cytokines is mainly through parasite, not dysbiosis. In summary, microbiota dysbiosis may facilitate the development of depression by increasing the release of pro-inflammatory cytokines and impairing the Treg/Th17 balance **(** **Figure 3** **)**.

### Microbial metabolites

A substantial body of evidence supports that SCFAs play a pivotal role in the development of depression. The levels of acetic acid and isocaproic acid are significantly lower in the depressive women than the matched healthy subjects (Skonieczna-Żydecka et al., 2018). Moreover, the concentrations of acetate and propionate are negatively associated with the depression score (Skonieczna-Żydecka et al., 2018), indicating that circulating SCFA levels might serve as an indicator of the severity of depression. SCFAs can be transferred through BBB (Kekuda et al., 2013; Vijay and Morris, 2014) and fortify the BBB integrity by regulating the expression of tight junction proteins (Braniste et al., 2014). Although the mechanistic evidence on how SCFAs modulate neurological disorders is not well-documented, several signaling pathways including the peroxisome proliferator-activated receptor gamma (PPARγ) pathway and the Nuclear factor erythroid 2-related factor 2 (Nrf2) redox pathway might be involved, since the activation of both signaling pathways are related to the alleviation of depression and stress (Yao et al., 2016; Gold, 2021).

## Effects of probiotics on perinatal depression

Recently, probiotics have been used to modulate the gut microbiome and improve cognition, as they target the MGBA. Considering the impact of microbiota dysbiosis on mental health disorders including anxiety and depression, probiotic intake may be an effective strategy for preventing and alleviating perinatal depression.

**Figure 4:**
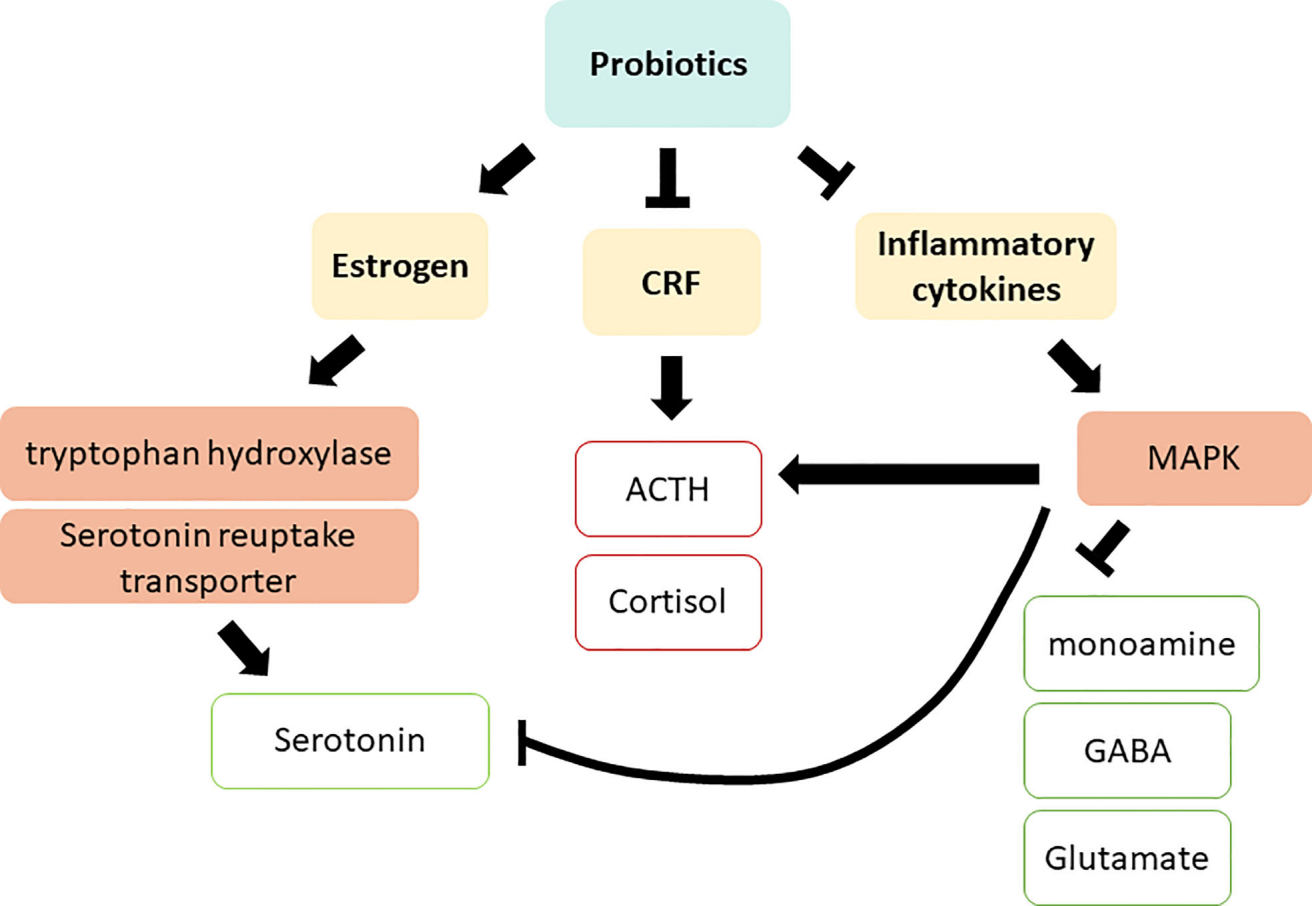
Action mechanisms of probiotics on depression *via* regulating systemic biomarkers. CRF, Corticotropin releasing factor; ACTH, adrenocorticotropic hormone; MAPK, MAP kinase; GABA, γ-Aminobutyric acid. Red squares indicate an increased level in depressive patients; green squares indicate a decreased level in depressive patients.

Probiotics can modulate depression by altering the systemic biomarkers discussed in the previous section. Several *in vivo* studies have revealed that supplementation with *Lactobacillus* probiotics promotes estrogen levels and alleviates diseases resulting from reduced estrogen levels (Itoh et al., 2011; Britton et al., 2014; Guo et al., 2016). In a clinical trial on postmenopausal osteopenic women, probiotic and isoflavone aglycone supplementation significantly enhanced the ratio of urinary 2-hydroxyestrone (2-OH) to 16α-hydroxyestrone (16α-OH), favoring estrogen metabolite profile (Lambert et al., 2017). In addition to reproductive hormones, probiotics also regulate stress hormones. Yang et al. suggested that probiotic supplementation effectively enhanced serum CRF levels, thereby relieving anxiety in patients before surgery (Yang et al., 2016). Similarly, in rats, oral administration of *L. farciminis* substantially decreased circulating ACTH and corticosterone, as well as reduced hypothalamic CRF and pro-inflammatory cytokine expression (Ait-Belgnaoui et al., 2012). In mice, the administration of lactic acid bacteria including *B. longum subsp. infantis E41* and *B. breve M2CF22M7* substantially enhanced the secretion of 5-hydroxytryptophan (5-HTP) and mitigated depressive behavior (Tian et al., 2019). Furthermore, oral administration of *Bifidobacterium* altered serotonin metabolism in the brain stem (Desbonnet et al., 2008). Although diet is a well-known determinant of the gut microbiota profile, a study on rats indicated that probiotic supplementation markedly mitigated depression and skewed the production of pro-inflammatory cytokines, independent of diet (Abildgaard et al., 2017). These studies suggest the efficacy of probiotic supplementation in altering reproductive hormones, stress related hormones, and inflammatory cytokines, all of which are closely linked to the development of depression **(** **Figure 4** **)**, as discussed in section 2.

In humans, the consumption of a beverage high in *L. casei* strain *Shirota*, *B. breve* strain Yakult, *L. lactis*, and *Streptococcus thermophilus*, twice a day for 28 days significantly improved stress resilience and reduced anxiety (Kato et al., 2021). Mi et al. revealed that *L. reuteri* supplementation at a dose of 10^8^ [colony-forming units (CFU)] significantly reduced infant colic and mitigated maternal depression during infantile colic (Mi et al., 2015).

Nevertheless, the effects of probiotics on mitigating perinatal depression have been controversial. A study by Slykerman et al. on 423 females, who were at 14-16 weeks pregnant and received either placebo or *L. rhamnosus* HN001 (6 x 10^9^ CFU/g) from enrolment until six-months postpartum indicated that probiotic supplementation significantly reduced depression and anxiety scores during the postpartum period (Slykerman et al., 2017a). However, using the same *L. rhamnosus* strain, in addition to *B. lactis*, Dawe et al. did not observe clinically significant effects of the probiotic mixture (6.5 × 10^9^ CFU) on anxiety and mental health scores of 230 obese pregnant women y (Dawe et al., 2020). Similarly, a recent clinical trial (n = 40) reported that the intake of a probiotic mixture of several species (2.5 × 10^9^ CFU; *B. bifidum*, *B. lactis*, *L. acidophilus*, *L. brevis*, *L. casei*, *L. salivarius*, and *L. lactis*) did not effectively alleviate maternal stress and anxiety, or improve the mother-infant bonding (Browne et al., 2021). In contrast, in another clinical trial on overweight and obese women, daily probiotic supplementation (*L. rhamnosus* and *B. animalis ssp. Lactis*; each 10^10^ CFU) increased depression scores at 12-months postpartum **(** **Table 3** **)**. Therefore, whether probiotics are beneficial for perinatal depression is still debatable.

**Table 3:** Characteristics of studies evaluating the role of probiotics in perinatal depression.

Author, year	Number of subjects	Intervention period	Probiotics	Result
Browne, 2021	Probiotics (n = 20); placebo (n = 20).	From 26 to 30 weeks of gestation until delivery.	*B. bifidum* W23, *B. lactis* W51, *B. lactis* W52, *L. acidophilus* W37, *L. brevis* W63, *L. casei* W56, *L. salivarius* W24, *L. lactis* W19 and *L. lactis* W58	Probiotics significantly enhanced cognitive reactivity but did not alter anxiety (general, maternal), depression, and pregnancy-related and general daily hassles.
Hulkkonen, 2021	Fish oil (FO) + placebo (n = 87); probiotics + placebo (n = 96); FO + probiotics (n = 93); placebo + placebo (n = 67).	From <18 weeks of gestation until 6-month postpartum.	*L. rhamnosus* HN001 and *B. animalis* ssp. *lactis* 420	Compared with the FO + placebo group, FO + probiotics group had a significantly higher depression score from early to late pregnancy.
Dawe, 2020	Probiotics (n = 115); placebo (n = 115).	From 12-18 weeks of gestation until 36 weeks of gestation	*L. rhamnosus* GG and *B. lactis* BB12	Probiotics did not improve maternal anxiety, and functional health and well-being (physical and mental).
Slykerman, 2017	Probiotics (n = 212); placebo (n = 211).	From 14-16 weeks of gestation until 6-month postpartum	*L. rhamnosus* HN001	Probiotic supplementation was significantly associated with reduced depression and anxiety.

## Discussion

Extensive evidence suggest that nutritional and dietary interventions are associated with perinatal depression, as changes in dietary habits and nutrition can alter microbiota profile within a short period (Klimenko et al., 2018). Therefore, microbiota may modulate the efficacy of nutritional and dietary interventions on perinatal depression.

Despite sevαal studies on the mechanism by which the gut microbiota might impact mood and cognition, particularly by targeting the MGBA, the role of probiotic supplementation in alleviating perinatal depression is unclear. There is also a dearth of clinical trials describing how probiotic interventions may mitigate maternal depression, anxiety, and arousal.

Although several studies have reported the benefits of probiotic supplementation for reducing antenatal depression, they have been limited by the absence of any significant effects. Firstly, the relatively small sample size and short intervention period reduced the potential to observe a statistically significantly difference between the control and the supplemented groups. Secondly, most of the participants exhibited a high socio-economic status and healthy lifestyle (less smoking), suggesting that they might have undergone less stressful situations in daily life, and were more aware of their mental and psychological health. This might diminish the difference between the treatment and control groups.

Reports indicate that different probiotic strains may differently regulate mood and behavior. For example, colonization with *B. infantis* inhibited locomotor activity and did not affect anxiety levels in mice (Nishino et al., 2013; Tian et al., 2019), whereas monoassociation with *Brautia coccoides* significantly decreased anxiety levels, with only a slight impact on locomotor activity (Nishino et al., 2013).

In conclusion, preventing or alleviating perinatal depression by targeting the MGBA using probiotic supplements is a promising approach. However, increased studies are warranted to validate the efficacy of certain probiotic strains in antenatal and postnatal depression and depression-related behavioral changes. Thus, further investigations on this theme will enable suggesting recommendations and guidelines on probiotic supplementation to mitigate perinatal depression.

## Author contributions

JS, BZ, YY and XL designed the study. JS, BZ, JK and GL reviewed literatures. SZ, LS, XZ, XY, JM, and JC extracted information. JS, BZ, JK, GL, SZ, LS and XZ wrote the first draft of the manuscript. XY, JM and JC provided figures. All the authors revised the manuscript and provided the final version of the manuscript.

## Conflict of interest

Author JC was employed by company Ingredion Incorporated.

The remaining authors declare that the research was conducted in the absence of any commercial or financial relationships that could be construed as a potential conflict of interest.

## Publisher’s note

All claims expressed in this article are solely those of the authors and do not necessarily represent those of their affiliated organizations, or those of the publisher, the editors and the reviewers. Any product that may be evaluated in this article, or claim that may be made by its manufacturer, is not guaranteed or endorsed by the publisher.
