# Corrigendum: Gut microbiota: Linking nutrition and perinatal depression

**DOI:** 10.3389/fcimb.2022.1053553

**Published:** 2022-11-11

**Authors:** Jia Song, Bi Zhou, Juntao Kan, Guangya Liu, Sheng Zhang, Liang Si, Xianping Zhang, Xue Yang, Junhua Ma, Junrui Cheng, Xiaobo Liu, Yongde Yang

**Affiliations:** ^1^ Affiliated Wuhan Mental Health Center, Tongji Medical College, Huazhong University of Science and Technology, Wuhan, China; ^2^ Nutrilite Health Institute, Shanghai, China; ^3^ Jinyintan Hospital, Wuhan, China; ^4^ Ingredion Incorporated, NJ, United States

**Keywords:** fiber, perinatal depression, nutrition, microbiota, probiotics

## Incorrect affiliation

In the published article, there was an error in Xue Yang’s affiliation. Instead of “^2^Nutrilite Health Institute”, it should be “^1^Affiliated Wuhan Mental Health Center, Tongji Medical College, Huazhong University of Science and Technology, Wuhan, China”.

The authors apologize for this error and state that this does not change the scientific conclusions of the article in any way. The original article has been updated.

## Error in author list

In the published article, there was an error in the author list, and author order Yongde Yang and Xiaobo Liu was erroneously switched. The corrected author list appears below.

Jia Song^1^†, Bi Zhou^1^†, Juntao Kan^2^, Guangya Liu^3^, Sheng Zhang^1^, Liang Si^1^, Xianping Zhang^1^, Xue Yang^1^, Junhua Ma^1^, Junrui Cheng^4^, Xiaobo Liu^1*^ and Yongde Yang^1*^

The order of the Correspondence should be Yongde Yang as the first correspondence and Xiaobo Liu as the second correspondence.

The authors apologize for this error and state that this does not change the scientific conclusions of the article in any way. The original article has been updated.

## Publisher’s note

All claims expressed in this article are solely those of the authors and do not necessarily represent those of their affiliated organizations, or those of the publisher, the editors and the reviewers. Any product that may be evaluated in this article, or claim that may be made by its manufacturer, is not guaranteed or endorsed by the publisher.

